# Exercise enhances: study protocol of a randomized controlled trial on aerobic exercise as depression treatment augmentation

**DOI:** 10.1186/s12888-020-02989-z

**Published:** 2020-12-09

**Authors:** Michèle Schmitter, Jan Spijker, Filip Smit, Indira Tendolkar, Anne-Marie Derksen, Peter Oostelbos, Ben F. M. Wijnen, Tessa J. van Doesum, Jasper A. J. Smits, Janna N. Vrijsen

**Affiliations:** 1 Depression Expertise Centre, Pro Persona Mental Health Care, Nijmegen, The Netherlands; 2 Behavioural Science Institute, Radboud University Nijmegen, Nijmegen, The Netherlands; 3 Trimbos Institute (Netherlands Institute of Mental Health and Addiction), Utrecht, The Netherlands; 4 Department of Epidemiology and Biostatistics & Department of Clinical Psychology, Amsterdam Public Health Research Institute, University Medical Centers Amsterdam (location VUmc), Amsterdam, The Netherlands; 5 Department of Psychiatry, Radboud University Nijmegen, Donders Institute for Brain, Cognition and Behaviour, Nijmegen, The Netherlands; 6 GGNet Network for Mental Health Care, Zutphen, The Netherlands; 7 Dutch Depression Association, Amersfoort, The Netherlands and De Hartenboom, Randwijk, The Netherlands; 8 Department of Clinical Epidemiology and Medical Technology Assessment, Maastricht University Medical Center, Maastricht, The Netherlands; 9 Department of Psychology & Institute for Mental Health Research, University of Texas at Austin, Austin, TX USA

**Keywords:** Exercise, Treatment augmentation, Depression, RCT, (cost-)effectiveness

## Abstract

**Background:**

Major depressive disorder (MDD) is a considerable public health concern. In spite of evidence-based treatments for MDD, many patients do not improve and relapse is common. Therefore, improving treatment outcomes is much needed and adjunct exercise treatment may have great potential. Exercise was shown to be effective as monotherapy for depression and as augmentation strategy, with evidence for increasing neuroplasticity. Data on the cost-effectiveness and the long-term effects of adjunct exercise treatment are missing. Similarly, the cognitive pathways toward remission are not well understood.

**Methods:**

The present study is designed as a multicenter randomized superiority trial in two parallel groups with follow-up assessments up to 15 months. Currently depressed outpatients (*N* = 120) are randomized to guideline concordant Standard Care (gcSC) alone or gcSC with adjunct exercise treatment for 12 weeks. Randomization is stratified by gender and setting, using a four, six, and eight block design. Exercise treatment is offered in accordance with the NICE guidelines and empirical evidence, consisting of one supervised and two at-home exercise sessions per week at moderate intensity. We expect that gcSC with adjunct exercise treatment is more (cost-)effective in decreasing depressive symptoms compared to gcSC alone. Moreover, we will investigate the effect of adjunct exercise treatment on other health-related outcomes (i.e. functioning, fitness, physical activity, health-related quality of life, and motivation and energy). In addition, the mechanisms of change will be studied by exploring any change in rumination, self-esteem, and memory bias as possible mediators between exercise treatment and depression outcomes.

**Discussion:**

The present trial aims to inform the scientific and clinical community about the (cost-)effectiveness and psychosocial mechanisms of change of adjunct exercise treatment when implemented in the mental health service setting. Results of the present study may improve treatment outcomes in MDD and facilitate implementation of prescriptive exercise treatment in outpatient settings.

**Trial registration:**

This trial is registered within the Netherlands Trial Register (code: NL8432, date: 6th March, 2020).

## Background

Around 268 million people worldwide suffer from major depressive disorder (MDD), indicating that depression is a highly prevalent condition [1]. Those affected experience persistent feelings of sadness and a loss of pleasure or interest in activities. Physical comorbidities such as cardiovascular diseases [2] and metabolic risk factors [3] are common too and MDD is one of the leading causes of disability and mortality worldwide [4]. Moreover, MDD is not just a burden for the individual, but also impacting the society as a whole. For example, incremental costs of patients suffering from MDD have been estimated at $210 billion in the United States [5]. Given the high prevalence and personal and societal burden, improving treatment of MDD is essential.

Standard treatment options consist of antidepressant medication and/or psychological interventions such as cognitive behavior therapy (CBT) and interpersonal therapy (IPT). While those treatment options are overall effective and generally of comparable effectiveness [6], approximately 40% of depressed patients do not respond adequately to treatment [7, 8] and recurrence rates are high (i.e. greater than 60%) [9]. Importantly, most treatment options have disadvantages, including high costs and unpleasant side effects. Moreover, they have no direct effect on somatic risk factors and comorbid somatic diseases. Hence, there is a need for new (combination) treatments.

One such treatment option is physical exercise. Exercise has been shown to be effective as monotherapy [10–13] and as augmentation strategy for antidepressant medication and psychological treatment [14–19]. Previous research showed that especially the combination of exercise and another treatment (i.e. antidepressants or psychological intervention) is effective in reducing depressive symptoms [19, 20]. Further, exercise may prevent relapse [21], benefits physical comorbidities [22], and is - unlike other treatments for depression - inexpensive, universally accessible, and shows favourable tolerability [23]. Hence, implementing an evidence-based exercise treatment module within depression standard care seems feasible and is hypothesized to be (cost-)effective. Nonetheless, the long-term effects of exercise treatment are largely unexplored [24, 25]. Therefore, the first aim of the present study is to investigate the (cost-)effectiveness of exercise as adjunct treatment for depression within the mental health system, including examination of the long-term effects up to 15 months.

Furthermore, exercise might not only improve depression but might benefit other health outcomes as well. For instance, regular exercise leads to improved fitness [26, 27] and several studies showed that exercise programs increase overall physical activity levels [28–30]. Similarly, exercise interventions are used to improve physical functioning in the elderly [31] and to counteract fatigue in somatic patients [32]. These positive effects can also be expected in depressed individuals engaging in regular exercise [33, 34]. Therefore, the second aim of this study is to examine the effect of exercise treatment on additional (mental) health outcomes, focusing on fitness, physical activity, health-related quality of life, disability, and motivation and energy.

Most research has focused on the effectiveness of exercise treatment for depression, with increasing but still limited attention for its mechanisms of change. Recently, Kandola and colleagues [35] reviewed evidence for the biological and psychosocial working mechanisms of physical activity and exercise specifically. They report evidence for increased neuroplasticity, especially in the hippocampus which is relevant to cognitive emotional processing and memory functions [35–37]. The psychosocial mechanisms are less well understood, with some research pointing toward self-esteem, social support, and self-efficacy [35, 38]. The authors therefore conclude that especially research on cognitive and psychosocial mechanisms is desirable. Knowing how an intervention works is essential to improving intervention techniques. Therefore, the third aim of the present study is to investigate cognitive and psychosocial mechanisms of change, focusing on rumination, self-esteem, and memory bias.

Rumination is a common symptom of depression and associated with its onset, maintenance, and recurrence [39]. Studies showed that exercise not only affects depressive symptoms in general, but also decreases rumination [40, 41]. Those studies indicate that rumination may mediate the effect of exercise on depressive symptoms. Moreover, exercise increases self-esteem and initial findings suggest that self-esteem mediates the relation between exercise and depression [35]. We aim to replicate the evidence for rumination and self-esteem as possible mechanisms of change. In addition, we examine memory bias as potential mediator which is related to rumination [42, 43] and self-esteem [44]. Negative bias in recall of self-related information has been recognized as important factor in the development and maintenance of depression (see cognitive model by Beck) [45–49]. As previous research identified that neuroplasticity in the hippocampal circuit increases through exercise which is linked to memory bias [50], changes in this circuit may allow depressed individuals to process emotional information differently, possibly decreasing the automatic preferential recall of negative information and in turn depressive symptoms. In summary, the aim of the present study is threefold: 1) we will investigate the long-term (cost-)effectiveness of adjunct exercise treatment and 2) its effect on additional positive outcomes as well as, 3) possible mechanism of change, focusing on rumination, self-esteem, and memory bias.

## Methods

### Study design

In this pragmatic superiority trial, patients are randomly assigned to either gcSC or the exercise intervention adjunct to gcSC. Measurements take place at baseline (T0), after session 3 (after 3 exercise sessions or 3 weeks of gcSC alone; T1), 6 (T2), 9 (T3) and 12 (T4), and 6 (T5), 9 (T6), 12 (T7), and 15 (T8) months post-baseline. Patients in the gcSC condition can receive the adjunct exercise intervention after completion of the follow-up assessments.

### Aims and hypotheses

1. The primary aim of the study is to evaluate the (cost-)effectiveness of exercise as adjunct treatment for depression in a randomized controlled trial (RCT). We expect that gcSC with the adjunct exercise intervention is more effective and cost-effective than gcSC alone in reducing depressive symptoms in outpatients with MDD, treated in Dutch specialised mental health services.
2. The second aim is to assess the effect of adjunct exercise treatment on physical activity, fitness, health-related quality of life, disability and motivation and energy. We hypothesize that gcSC plus adjunct exercise outperforms gcSC alone on these outcomes.
3. The third aim is to examine putative cognitive mechanisms of change. We expect that a reduction in ruminative thinking style, as well as, an increase in self-esteem and a decline in negative memory bias mediate the between-group effects on depression symptom severity.

### Participants

We will include 120 adult outpatients diagnosed with MDD, according to the Diagnostic and Statistical Manual of Mental Disorders [51]. Exclusion criteria are: Below the age of 16, lifetime manic episode, current psychosis, chronic depression (i.e. current depressive episode > 2 years), dysthymic disorder, high health risks of physical activity based on the screening tool Physical Activity Risk Questionnaire (PAR-Q) [52], insufficient comprehension of the Dutch language to fill out the questionnaires, and physical, cognitive, or intellectual impairments interfering with participation or informed consent.

### Sample size calculation

A recent meta-analysis on the effectiveness of exercise interventions compared to non-exercise comparators in adult outpatients with MDD [10] reports an overall medium to large effect on depressive symptoms, with Hedges’s g = 0.79. This meta-analysis included 11 trials, investigating both exercise as adjunct treatment to gcSC and exercise as monotherapy for depression. In order to provide the most optimal effect size estimate, we conducted our own meta-analysis, including only the trials examining the augmenting effect of exercise, to synthesize the available evidence from the most comparable trials. We identified four studies (moderate/high level of evidence; GRADE) [53] with similar designs to the present RCT that evaluated aerobic exercise as adjunct to gcSC in unipolar depressed mental health care outpatients, with depressive symptom levels as outcome [54–57]. The meta-analysis yielded a Hedge’s g effect size of 0.98 (95%CI = 0.51 ~ 1.45), se = 0.26, *t* = 3.84, *p* = .032, with only moderate heterogeneity, I^2^ = 37%. One study by Rueter and colleagues [56] appeared an outlier (based on the Galbraith plot). When removing this study to account for variations across studies, heterogeneity disappeared with I^2^ = 0%, while the effect size was similar to the original meta-analysis, including 11 trials (g = 0.79; 95%CI 0.42 ~ 1.17; se = 0.190; *p* = .053). Based on the latter, we assumed a between-group effect size of g = 0.70 for the power analysis.

The power calculation was based on guidelines offered by Hemming et al. [58] and accounted for the hierarchical data structure with patients being clustered in psychomotor therapists with a mean cluster size of *N* = 10 (range 5 ~ 15) [59] and an intra-cluster correlation of 0.05 [60], which introduces a design effect of 1.48. In addition, we assumed a correlation between baseline depressive symptoms and the relevant follow-up of *r* = 0.30 for the planned baseline-adjusted multilevel mixed analysis. In order to be able to detect an effect size g ≥ 0.70 as statistically significant at α < 0.05 (two-sided) with a power of (1-β) = 0.80, power analyses revealed a required minimum *N* = 60 per arm (*N* = 120 in total).

### Procedure

**Fig. 1:**
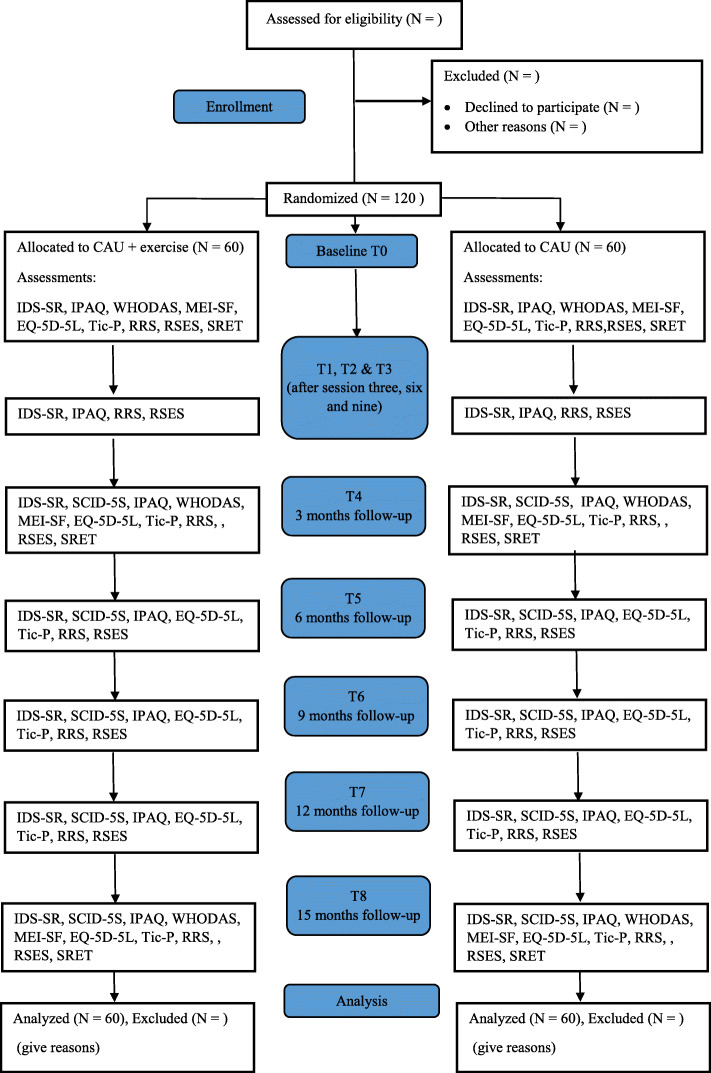
CONSORT flow chart

The current study takes place within the Dutch specialized mental health care setting and is a multicenter trial. Patients are recruited via Pro Persona mental health care (four locations: Arnhem, Ede, Nijmegen, Tiel), the Radboud University Medical Center (Nijmegen), and GGNet Network for mental health care (location Zutphen). If patients are eligible for participation, the therapists approach them for the study and they are contacted by a researcher. Study information is provided via a letter. Patients are contacted again after at least 48 h to discuss participation and answer any remaining questions. If patients decide to participate, they are asked to sign the informed consent form and are then randomized to either the gcSC condition or the gcSC with adjunct exercise treatment condition. The participant flow is depicted in Fig. 1.

Patients in the gcSC with adjunct exercise condition start the exercise treatment simultaneously to the gcSC treatment (with a maximum two-week difference between start dates). Patients complete the baseline assessment in the week before the first treatment session. The surveys are sent via email using the online cloud-based data solution program, Castor EDC and can be filled out via any personal device (e.g. computer, tablet). This program is also used for the Case Report Forms and initial data management. Additionally, patients receiving the adjunct exercise treatment fill out a short questionnaire before and after each supervised exercise session on paper, as part of the treatment. An overview of the assessments can be found in Table 1 and Fig. 1. Expected study duration is 36 months. Inclusion of patients started in March 2020.

**Table 1:** Assessment schedule

		Time point								
Endpoints	Material	T0	T1	T2	T3	T4	T5	T6	T7	T8
Primary outcome	IDS-SR	X	X	X	X	X	X	X	X	X
Secondary outcomes	SCID-5-S					X	X	X	X	X
	IPAQ	X	X	X	X	X	X	X	X	X
	Fitness^a^	X				X				
	WHODAS	X				X				X
	MEI-SF	X				X				X
Economic evaluation	EQ-5D-5L	X				X	X	X	X	X
	Tic-P	X				X	X	X	X	X
Mechanisms	RRS	X	X	X	X	X	X	X	X	X
	RSES	X	X	X	X	X	X	X	X	X
	SRET	X				X				X

^a^Assessed in 15% of randomly selected patients from both conditions

*IDS-SR* Inventory of Depressive Symptomatology (i.e. depressive symptoms)*, SCID-5-S* Structured Clinical Interview for DSM-5 (i.e. depressive remission), *IPAQ* International Physical Activity Questionnaire (i.e. physical activity), *WHODAS* World Health Organization Disability Assessment Schedule (i.e. disability), *MEI-SF* Motivation and Energy Inventory-Short Form (i.e. motivation and energy), *EQ-5D-5L* (i.e. quality of health), *Tic-P* Trimbos Institute and iMTA Cost questionnaire for Psychiatry (i.e. economic costs), *RRS* Ruminative Response Scale (i.e. rumination), *RSES* Rosenberg Self-Esteem Scale (i.e. self-esteem), *SRET* Self-Referent Encoding Task (i.e. memory bias)

### Ethics

The present study was approved by the Medical Ethics Committee of Arnhem-Nijmegen (under the registration code: NL72080.091.19) and is registered within the Netherlands Trial Register (code: NL8432). Because recently self-esteem was found to be a possible mechanism of change of exercise treatment [35], we decided to include a self-esteem measure after preregistration. All data will be processed in a confidential manner and data is processed using an identification code with only the principal investigator (PI) and the coordinating investigators (CI) having access to the key. The research process is regularly monitored by a trained and independent monitor, which includes monitoring of the completeness of informed consents, data safety, and correspondence with the ethics committee. Serious adverse events will be reported to the Medical Ethics Committee, although none are expected due to the low-risk intervention.

### Randomization

Randomization is stratified for gender and setting, using Castor EDC. This program consecutively enrolls patients in line with a four, six and eight block design. These blocks are created for both strata separately. The first block is generated with the first randomization and thereafter a new block is randomly selected and generated when the previous one is used up. Allocation of patients is randomly selected from the block in use. More information on the randomization algorithm can be found on the website of Castor EDC.^1^

### Interventions

***gcSC.*** The gcSC treatment is delivered in line with the Dutch multidisciplinary guidelines for depression treatment, consisting of pharmacological and/or psychological interventions which are offered individually or in group format.^2^ The gcSC condition may also receive some form of body-oriented or physical activity treatment but no prescribed exercise treatment.

#### Adjunct exercise

Patients receiving the adjunct exercise treatment, exercise once a week under supervision of psychomotor therapists or trained nurses and commit to exercising twice a week at home for 12 weeks. Supervised exercise generally consists of running or indoor cycling (spinning), which are usually both offered in a group setting, although other exercise types and individual supervised exercise are also provided. The exercise sessions last for 45 min at moderate intensity, which is based on heart rate frequency (i.e. *moderate = 64–76% of HRmax = 220 – age)*. This kind of exercise intervention is evidence-based and recommended by the National Institute for Health and Care Excellence (NICE) [61]. Patients track the intensity, duration, and frequency of each exercise session, with the aid of a non-invasive activity tracker (Fitbit)^3^ which is provided for the length of the study (i.e. 15 months).

### Material

#### Depressive symptoms

To assess the severity of depressive symptoms, as primary outcome of the RCT, the Dutch version of the Inventory of Depressive Symptomatology-Self Report (IDS-SR) [62] is used. This scale consists of 30 items, measuring the severity of different depressive symptoms on a four-point Likert scale. Validity and reliability have been established, with Cronbach’s alpha ranging between 0.76 and 0.82 in depressed outpatients [62].

#### Remission

The clinician-rated Structured Clinical Interview for DSM-5 (SCID-5-S) [63] is used to assess clinically defined remission at follow-up. The SCID-5-S is administered over the phone by trained and blinded assessors, conform recommendations [25]. The Dutch version was shown to have moderate to excellent inter-rater agreement of the Axis I disorders [64].

#### Physical activity and fitness

To assess self-rated physical activity level, the Dutch version of the International Physical Activity Questionnaire (IPAQ) [65] is used. Psychometric properties of this questionnaire were shown to be acceptable in different settings and populations [66]. Moreover, to assess objective physical activity level and heart rate, patients receive the Fitbit in the adjunct exercise intervention condition, as it allows self-dosing during the exercise sessions, enables patients to learn how to effectively exercise to counteract depression, and helps patients motivate themselves, hence contributing to adherence. Additionally, to objectively assess physical fitness, fitness tests will be conducted at baseline and after the 12 weeks of exercise or gcSC treatment in 15% of the sample (selected at random). Here, the main endpoint is improvement in cardiorespiratory fitness.

#### Mood

Patients provide a positive and negative mood rating (on Visual Analogue Scales) directly before and after each supervised exercise session. They are encouraged to also use the mood ratings at home to monitor direct mood benefits. This is a technique used in clinical practice, to motivate patients to engage in exercise [67] and can thereby contribute to adherence and long-term exercise [68].

#### At-home exercise

Embedded in the same questionnaire as the mood ratings, patients indicate the type, frequency, duration, and intensity (mean and peak heart rate, as indicated by the Fitbit) of the previous two weekly at-home sessions. The psychomotor therapists/nurses discuss how the week went and together with the patient adapt the personalized exercise plan if needed. To motivate patients, different strategies are used that are integrated in the personalized plan. These strategies are based on previous research [67, 68] and psychomotor clinical practice and include psychoeducation, weekly exercise session scheduling, and goal setting.

#### Disability

Disability is assessed with the Dutch version of the World Health Organization Disability Assessment Schedule (WHODAS 2.0) [69]. Thirty-six items are designed to measure functioning in different domains on a five-point Likert scale, ranging from 0 (i.e. no effort at all) to 4 (i.e. much effort). Reliability and validity of the scale are established for different populations [69].

#### Motivation and energy

To assess motivation and energy, the Motivation and Energy Inventory-Short Form (MEI-SF) [70] is used. The MEI-SF was translated into Dutch and then back-translated into English by bilinguals from the University of Texas at Austin. On 18 items, this scale measures the level of motivation and energy using seven-point Likert scales, ranging from 0 (i.e. none of the time) to 6 (i.e. all the time). This scale was specifically developed to evaluate interventions designed to improve motivation and energy in depressed patients. In previous research, Cronbach’s alpha ranged between .86 and .94 at baseline and .95 and .96 at the end of study, indicating high internal consistency [70].

#### Health-related quality of life

For the health-economic evaluation, changes in patients’ health-related quality of life are assessed with the EuroQol’s 5-level version, the EQ-5D-5L (https://euroqol.org) in combination with the Dutch tariff [71]. Patients indicate their health state on different domains (i.e. mobility, self-care, usual activity, pain/discomfort and anxiety/depression), which is then transformed into a health state valuation (a utility score) which is anchored between 0 (i.e. dead) and 1 (i.e. perfect health). The utility is used to calculate the quality-adjusted life-years (QALY) over the studies follow-up time. Hence, the length of time spend in a particular health state is weighed by the utility score to obtain the QALY.

#### Healthcare costs

and costs stemming from productivity losses are assessed with the Trimbos Institute and iMTA Cost questionnaire for Psychiatry (Tic-P) [72] which measures resource use such as use of health services, patients’ and their family’s out-of-pocket costs, and lesser productivity owing to absenteeism and work cutback (presenteeism).

#### Rumination

Rumination is measured using the Dutch version of the Ruminative Response Scale (RRS) which was shown to be a valid and reliable tool for this purpose [73]. This scale consists of 26 items which are rated on four-point Likert scale, ranging from 0 (i.e. almost never) to 3 (i.e. almost always).

#### Self-esteem

Self-esteem is assessed using the Dutch version of the Rosenberg self-esteem scale (RSES) [74]. Global self-esteem is measured with ten items on a four-point Likert scale, ranging from 0 (i.e. strongly disagree) to 3 (i.e. strongly agree). Psychometric properties of the Dutch version were established, with a one-factor solution and high internal consistency [75].

#### Memory bias

To measure memory bias, the computerized Self-Referent Encoding Task (SRET) [76] is used. This task is a reliable method to assess memory bias [76, 77]. During the encoding phase, twelve positively and twelve negatively valenced adjectives are presented on the computer screen, one by one. No more than two words of the same valence are presented sequentially. Patients make a categorical decision whether the adjective is self-descriptive or not, by pressing on either of two keyboard buttons. After a 2 min distraction task (i.e., the Trail Making Test), the patients have 3 min to type in all the words they can remember from the previous task. Spelling errors are permitted.

### Statistical analysis

Given the ongoing advances in statistics and technology, all analyses will be performed in line with best current practice. Following the CONSORT statement [78], all our analyses will adhere to the intention-to-treat (ITT) principle (i.e., with an analysis of all patients as randomized). To this end, analyses of treatment effects will be conducted with multilevel mixed modelling, with treatment condition as factor and the depended measure as baseline covariate, while missing observations will be multiply imputed (with chained equations) for the health-economic analyses. In addition, we will also report the results in the per-protocol sample (≥ 6 supervised exercise sessions). Generalized (logit) mixed modelling will be used to compare the conditions on number of patients in remission at each follow-up measure. To examine whether rumination, self-esteem and memory bias are the mechanisms of change between adjunct exercise treatment and a decrease in depressive symptoms, multilevel structural equations modelling will be performed in Stata [79], or alternatively Hayes’ PROCESS macro [80] for SPSS [81] will be used.

Finally, a cost-effectiveness analysis (CEA) and cost-utility analysis (CUA) will be performed from a societal perspective [78]. We will consider four types of costs: 1) intervention costs, 2) healthcare costs, 3) patients’ and family out of pocket costs and opportunity costs of informal care, 4) productivity losses stemming from absenteeism and lesser efficiency while at work (presenteeism). The incremental cost-effectiveness ratio (ICER) will be computed to obtain the incremental costs per treatment responder and per QALY gained. Stochastic uncertainty will be handled using 2500 non-parametric bootstraps and by plotting the simulated ICERs on the ICER plane. For decision-making purposes, the ICER acceptability curve will be plotted for various willingness-to-pay (WTP) ceilings for making judgements whether the adjunctive exercise intervention offers good value for money relative to the care as usual comparator condition. One-way sensitivity analyses directed at uncertainty in the main cost drivers and outcomes will be performed to assess the robustness of our findings. Both the analysis and reporting of the findings will conform to the CONSORT [82], the Dutch guidelines for economic evaluations in health care, and CHEERS statements [78, 83]. All health-economic analysis will be conducted in R or Stata/SE 16.1 or later [79].

## Discussion

The aim of the present study is to examine the (cost-)effectiveness of adjunct exercise treatment in the outpatient treatment of depression. We will also investigate long-term effects up to 15 months and the effect of exercise on additional outcomes such as physical activity and fitness, disability, health-related quality of life and motivation and energy. Finally, we aim to examine potential mechanisms of change, focusing on the major cognitive and psychosocial processes contributing to depression i.e. rumination, self-esteem, and memory bias.

### Strengths and limitations

This study has several strengths. The RCT has the potential to provide a meaningful contribution to the literature on the cost-effectiveness and working mechanisms of an exercise prescription for the treatment of depression. The prospective study design enables us to follow patients for a period of 15 months post-baseline, to also examine the longer-term effects of exercise on depression. Finally, through the randomized design we will be able to compare whether adjunct exercise alongside usual care is more effective in decreasing depressive symptoms than gcSC alone. Because we provide the exercise intervention via psychomotor therapists and nurses who are already experienced in providing physical activity interventions, and do this within the naturalistic treatment setting, the RCT optimally facilitates later implementation of the evidence-based exercise protocol.

We further stimulate implementation in the (Dutch) mental health care system by flanking the RCT with a qualitative implementation study. The implementation study is two-folded. The first part will be used for scaling up and improving the implementation of an exercise augmentation prescription during the study. Psychomotor therapists/nurses, therapists who referred patients to the study, and patients who participated in the exercise treatment as well as patients who refused participation are interviewed to share their experiences. This allows for the identification of barriers and facilitating factors from an organisational level as well as an individual (patient) level. These results will be translated immediately to daily practice to enhance the implementation of prescribed exercise already during the RCT. The second part of the implementation study will identify facilitators and barriers for nationwide implementation once the current study shows (cost-)effective results. This will be based on (group-based) interviews with stakeholders who participated in the current research and for example with insurance companies and the clinical guidelines for depression treatment committee. The final results of the implementation study will be summarized in an implementation plan for future rollout.

Despite these strengths, there are some limitations that should be considered. First, patients in both conditions receive usual care in accordance with the Dutch multidisciplinary guidelines. While this reflects clinical practice, treatment heterogeneity in the gcSC is expected, as different psychological and/or pharmacological interventions are provided in different dosages and in varying formats (e.g. either group-based or individual CBT). Therefore, we can only draw general conclusions about the degree in which gcSC is augmented and merely explore which type of treatment is augmented most by exercise treatment. Nonetheless and due to the randomized design, the heterogeneity in gcSC should be similar across conditions, thus not thwart the study’s internal validity, while at the same time it increases the external validity of this pragmatic trial. Another benefit of a pragmatic trial is that future results can be optimally generalized and it facilitates the implementation of exercise in clinical practice, if proven (cost-)effective.

Other limitations are that exercise treatment is implemented as add-on treatment to gcSC which might lead to improvements in depressive symptoms due to nonspecific therapy effects such as more attention by therapists or peer support. The use of the Fitbit can also act as a co-intervention in the context of this trial, as it provides personal physiological feedback to patients in the exercise treatment condition. Finally, because patients decide whether they want to participate, there might be a selection bias due to the voluntary nature of the intervention. One may expect that patients who have a positive attitude toward exercise are more likely to participate. We register the reasons for refusal to participate and will explicitly invite patients who did not want to participate for our qualitative implementation study, in order to address the self-selection issue. Importantly, even if exercise treatment only attracts certain individuals, it can be an effective treatment module for depression, not limiting the additional value of this study in examining an accessible and acceptable treatment for depression.

### Clinical implications

Even though exercise as treatment for depression is proven effective and recommended in the NICE guidelines [61], it is generally not implemented following evidence-based guidelines, in Dutch (and international) clinical practice, neither is it included in educational programs of mental health care providers. This study will not only facilitate the implementation of prescribed exercise treatment but also aims to contribute to the much-needed improvement of MDD treatment outcome in an acceptable, effective, and cost-effective way.

## Data Availability

Not applicable.

## Abbreviations

MDD: Major depressive disorder
gcSC: guideline concordant Standard Care
WHO: World Health Organization
CBT: Cognitive behaviour therapy
IPT: Interpersonal therapy
RCT: Randomized controlled trial
APA: American Psychological Association
PAR-Q: Physical Activity Risk Questionnaire
PI: Principal investigator
CI: Coordinating investigators
IDS-SR: Inventory of Depressive Symptomatology-Self Report
SCID-5-S: Structured Clinical Interview for DSM-5
IPAQ: International Physical Activity Questionnaire
WHODAS: World Health Organization Disability Assessment Schedule
QALY: Quality-adjusted life-years
MEI-SF: Motivation and Energy Inventory-Short Form
Tic-P: Trimbos Institute and iMTA Cost questionnaire for Psychiatry
RRS: Ruminative Response Scale
RSES: Rosenberg self-esteem scale
SRET: Self-Referent Encoding Task
ITT: Intention-to-treat
CEA: Cost-effectiveness analysis
ICER: Incremental cost-effectiveness ratio

## Footnotes

1 For more information on the randomization algorithm of Castor EDC, see https://helpdesk.castoredc.com/article/50-the-randomization-algorithm-in-castor.

2 For the Dutch multidisciplinary guidelines we refer to the https://www.ggzstandaarden.nl/zorgstandaarden/depressieve-stoornissen.

3 Patients can either choose to use the Fitbit Alta HR or the Fitbit Charge 3. For more information on Fitbit, we refer to the website of the provider https://www.fitbit.com/de/home.
